# Oncologists' perceived barriers to an expanded role for primary care in breast cancer survivorship care

**DOI:** 10.1002/cam4.793

**Published:** 2016-06-30

**Authors:** Heather B. Neuman, Elizabeth A. Jacobs, Nicole M. Steffens, Nora Jacobson, Amye Tevaarwerk, Lee G. Wilke, Jennifer Tucholka, Caprice C. Greenberg

**Affiliations:** ^1^ Wisconsin Surgical Outcomes Research ProgramDepartment of SurgeryUniversity of Wisconsin School of Medicine and Public HealthMadisonWisconsin; ^2^ University of Wisconsin Carbone Cancer CenterUniversity of Wisconsin School of Medicine and Public HealthMadisonWisconsin; ^3^ Division of General Internal MedicineDepartment of MedicineUniversity of Wisconsin School of Medicine and Public HealthMadisonWisconsin; ^4^ Denver Public HealthDenver Health and Hospital AuthorityDenverColorado; ^5^ School of NursingInstitute for Clinical and Translational ResearchUniversity of Wisconsin‐MadisonMadisonWisconsin; ^6^ Department of MedicineDivision of Hematology and OncologyUniversity of Wisconsin School of Medicine and Public HealthMadisonWisconsin

**Keywords:** Breast cancer, cancer surveillance, cancer survivorship, primary care

## Abstract

Concern exists about the sustainability of traditional, oncologist‐led models of breast cancer survivorship care. However, many oncologists are hesitant about deferring survivorship care to primary care providers (PCPs). Our objective was to examine oncologists' perceptions of the role PCPs play in breast cancer survivorship and the rationale underlying these perceptions. One‐on‐one interviews with medical, radiation, and surgical oncologists in Wisconsin were conducted (*n* = 35) and transcribed. Data analysis was performed using an inductive approach to content analysis. Oncologist‐perceived barriers included: PCP's level of experience with cancer care; Lack of PCP comfort in providing survivorship care; Existing demands on PCPs' time; Patient preference for oncology‐led survivorship care. Oncologists described familiarity and trust in individual PCPs as factors that could mitigate barriers and lead to increased PCP involvement in survivorship care. Although a number of perceived barriers to PCP participation in survivorship were identified by Wisconsin oncologists, our findings support the direction of ongoing initiatives to facilitate PCP involvement. Our findings also suggest that early PCP involvement in survivorship may increase PCP comfort and patients' trust in PCPs in this role. The identified barrier most challenging to address may be the limited capacity of the current primary care system to manage follow‐up for breast cancer survivors.

## Introduction

Concern exists about the sustainability of traditional, oncologist‐led models of delivering breast cancer survivorship care in the face of the rapidly expanding number of breast cancer survivors and the projected shortfall of oncology providers 1, 2, 3. Shared survivorship care with primary care is one solution to this problem; it builds on the often already strong relationship between a patient and her primary care provider (PCP), ensures continuity for concurrent comorbid conditions, and improves quality of care as well as patient satisfaction with care 4, 5, 6, 7, 8, 9, 10, 11.

However, both PCPs and oncologists in the U.S. are hesitant about transferring primary responsibility for the cancer‐related components of survivorship care, such as surveillance for cancer recurrence, to PCPs. This hesitancy is particularly strong in the first 5 years after diagnosis 12, 13, 14, 15, 16. In a nationally representative sample of providers, only 16% of medical oncologists and 48% of PCPs preferred a shared or PCP‐led model of care 12, 13, 17, 18. The barriers to increasing primary care participation in breast cancer survivorship care are complex, and addressing them will require a multifaceted approach. However, as identified by Potosky, et al., an important step toward a shared‐care model is to further explore barriers to oncologists' willingness to share survivorship care responsibilities with PCPs” 12, 13. Several large survey‐based studies conducted to date have identified oncologists' attitudes toward PCP involvement as a barrier,^13^, ^14^, ^17^, ^18^, ^19^ but these studies are unable to provide sufficient insight into the reasons underlying the attitudes to identify opportunities to change behavior. Our objective is to address this gap in the literature by examining U.S. oncologists' perceptions of the role PCPs play in breast cancer survivorship care and the rationale underlying these perceptions.

## Methods

### Recruitment and data collection

Individual interviews were completed with medical, radiation and surgical oncologists who care for breast cancer patients in Wisconsin between May and October 2013. Maximum variation sampling was used to select a diverse sample of oncologists, including those who practiced in both community and academic‐based settings in different regions of the state, had varying years of experience, and whose practices varied in the percentage of breast cancer patients seen. We sent email invitations to potential participants, and identified additional participants through snowball sampling. For the purposes of this study, general surgeons who care for breast cancer patients were considered to be surgical oncologists. Participants were offered $75 for their time. The University of Wisconsin Health Sciences Institutional Review Board approved this study.

A trained interviewer (N.S) conducted one‐on‐one interviews (median 28 (19–48) min) using a semistructured interview guide. The interview guide included open‐ended questions designed to explore predetermined domains, with follow‐up questions used to expand or clarify participant responses. The interview guide was developed, piloted, and iteratively revised by the multidisciplinary research team. The interview covered areas relevant to survivorship care: what occurs during usual survivorship care visits, how oncologists perceived their own roles and responsibilities in breast cancer follow‐up, factors influencing oncologists' decisions to participate in ongoing survivorship care, and the perceived role PCPs played in survivorship care. Of the six primary questions included in the interview script, one focused specifically on the perceived role of PCPs in follow‐up. However, oncologists discussed PCPs at multiple other points in the interview, including during a case vignette where oncologists were asked to describe how follow‐up would be provided for a standard patient in their practice. Interviews were transcribed verbatim. Accrual continued until data saturation (i.e., the point at which no new themes were encountered) was achieved. We have previously published a manuscript focused on oncologists' perspectives on their own roles and responsibilities in follow‐up 20. The focus of the current analysis is on the role oncologists' perceive PCPs to play in survivorship care.

### Data analysis

Data collection and analysis occurred concurrently using an inductive approach to content analysis 21, 22. First, two investigators (H. N. and N. S.) performed open coding by independently reviewing 10 transcripts to develop an initial list of codes. The investigators then met to review each transcript and refine the initial code list into a preliminary coding taxonomy. All transcripts were reviewed independently by each investigator and coded using the preliminary coding taxonomy. The final transcript codes from each investigator were compared, and differences discussed and resolved through consensus. Throughout the study, the coding taxonomy was iteratively revised, adding, and removing codes as new concepts emerged; as changes were made, each interview was recoded to ensure consistency. Analysis continued until the primary codes were saturated and the coding taxonomy was stable. In the next stage of analysis, codes were grouped in conceptual categories that best represented the data. Selective and axial coding was performed in order to examine relationships between categories. Qualitative analysis software (NVIVO 10 software, QSR International Melbourne, Australia) was used to organize the data.

## Results

Thirty‐five interviews were completed with 12 medical, 11 radiation, and 12 surgical oncologists. Participating oncologists practiced in diverse practice settings (71% community‐based) and years of experience (median 11, range 2–48 years). Participants reported that breast cancer comprised a median 50% of their practices (range 10–100%). The majority of participants were male (57%).

### Oncologist‐identified barriers to PCP participation in survivorship care

The oncologists interviewed described a number of perceived challenges to PCP participation in survivorship care. The perceived barriers to PCP participation in survivorship care are summarized in Table 1. We elaborate on these themes and provide representative quotes below.

**Table 1:** Oncologists' perceived barriers to primary care provider participation in follow‐up care

Primary care providers (PCP's) level of experience with cancer care
Perceived limited training in survivorship care Challenging to remain current given quickly evolving nature of cancer care Oncologist concerns about PCP's ability to recognize and address issues related to cancer recurrence and late effects of treatment
Lack of PCP comfort in providing survivorship care
Lack of standard cancer follow‐up guidelines for PCPs to use Perceived preference by PCPs for oncologists to manage cancer follow‐up Perceived that PCP's are concerned about missing things
Existing demands on PCPs' time
Perceived lack of time in typical primary care visit to address survivorship issues Perceived lack of capacity in clinic for influx of new patients Perceived competing demands for PCP attention during short clinic visit Under‐reimbursement for primary care visits
Patient preference for oncology‐led survivorship care
Perceived lack of patient confidence in PCP breast exam Perception that patients receive more reassurance from oncologist

Oncologists recognized the importance of PCP involvement in the care of their breast cancer survivors; however, they perceived that PCPs' roles were limited to ongoing health maintenance or screening for other cancers, not as primary providers of survivorship care. The medical oncologists were generally more open than their surgical or radiation oncology colleagues to the idea of expanding PCPs' roles, given the similarities between their practices. As this medical oncologist shared:

Sometimes the private practitioner or the patient thinks that we have some sort of magic formula or something that we do that is super special and nobody else knows about, something secret that we can pick up cancer whereas they can't…but when you think about it, asking questions and examining people, that's not much different than primary care is doing.

#### PCP's level of experience with cancer care

Oncologists perceived that PCPs lack training in survivorship care and expressed concern about their level of experience with cancer care.

The vision would be that you would have well‐trained primary care physicians who had been trained in the [breast cancer treatment] process and effects and long‐term follow up of patients, so that they know what to look for and then turn [patients] over to [these PCP's] for the totality of care, but they would then be responsible for the cancer surveillance in an expert and effective manner. That group of physicians doesn't really exist right now.

The quickly evolving nature of cancer care was also a common concern regarding PCPs' ability to provide high‐quality survivorship care. Many of the oncologists in our study expressed the belief that it was challenging even for specialists to keep up with follow‐up recommendations and guidelines. Oncologists were specifically concerned about PCPs' ability to address issues related to cancer recurrence and late effects of treatment, with each oncology specialty voicing different concerns (Table 2).

**Table 2:** Oncologists' concerns related to cancer‐related components of survivorship care

Type of oncologist	Representative quote
Medical oncologist	If I could be satisfied that [PCPs] could reliably follow the treatment‐related guidelines for things like Tamoxifen and aromatase inhibitor, I'd also let them do that….I'm not perfectly satisfied that that's true, so I tend to take the primary responsibility for that.
Radiation oncologist	I think that the main benefit for radiation oncology staying involved is that a lot of patients don't know what is or isn't a radiation side‐effect and many nononcologists don't know those items either…I'm shocked at the number of things people attribute to radiation, which have nothing to do with it.
Surgical oncologist	My patients maintain their relationships with their primary care providers, but I do not want their primary care providers to be involved with their breast cancer treatment in follow‐up…'Cause they don't know how to do [a breast] exam.

#### Lack of PCP comfort in providing survivorship care

The oncologists also thought that many of the PCPs they interacted with were not comfortable and may not be particularly “*gung‐ho about taking the reins*” for survivorship care. They perceived that PCPs preferred oncologists maintain responsibility for the care of survivors.

The vast majority, at least in my experience with primary care docs, and I can't blame them, is they are so terrified that they're going to miss something‐ that they don't want to even deal with that responsibility and so regardless of what they're going to do, they still want the patient to see the oncologist, just kind of as the check point.

Oncologists thought that PCPs may be more willing to take responsibility for survivorship care for select subgroups of patients where the burden of following with an oncologist was high and/or the relative benefit of more intensive follow‐up for breast cancer was low. Specific examples cited included patients that must travel a long‐distance to see their oncologist or elderly patients with multiple comorbidities.

But I would also say that it's more like for a certain subgroup of patients. Let's say the 80–year‐old female patient who doesn't want much follow‐up anyway. You know, [PCPs] then say, “Well, I can just deal with that.” But I think for a younger patient, premenopausal, there are a lot of other issues, I think maybe, [PCPs] would probably not feel comfortable with that.

#### Existing demands on PCPs' time

Another challenge was the perceived burden of the primary care system workload and the limited capacity of the primary care system to absorb the additional care required by breast cancer survivors. Survivorship clinic visits were discussed as time consuming, with little overlap between what occurs during a typical primary care visit and what would be included in an optimal survivorship visit. Oncologists thought that “patients need time, especially the ones who've been through chemo and radiation and have potentially more significant issues and symptoms that need to get addressed.” Many oncologists expressed concern that PCPs may not be “paid enough and have time enough to handle a sudden influx*”* of breast cancer survivors into their practices. Furthermore, PCPs were perceived to have numerous competing demands for their attention during a clinic visit and an increasing number of benchmarks to meet. Medical oncologists, in particular empathized with how difficult it can be to balance the competing demands faced within the primary care setting.

The issue with primary care providers is that they're very pressured for time, because it's an under‐reimbursed type of care that's not specialized, so they have to take care of diabetes and hypertension and everything in that 15 minute appointment. I don't think they're incapable of doing [breast cancer follow‐up], I just think they're asked to do too many things to have perhaps the appropriate focus on this issue.

#### Patient preference for oncology‐led survivorship care

Finally, oncologists perceived that their patients preferred to have follow‐up with an oncologist. Patients were perceived to have greater confidence in an exam from their oncologist, and reassurance from an oncologist that cancer had not recurred was thought to carry more weight than a similar conversation with PCP.

I think, by and large, if they are scared about their cancer, they feel better after seeing any type of oncologist, surg, med, or rad. It may carry a little more weight, a little bit more panache, than a primary care doc.

### Factors that mitigate identified barriers

The oncologists participating in our study described familiarity and trust in PCPs as factors that could mitigate some of the barriers identified and lead to increased PCP involvement in survivorship care. Oncologists felt that they and the patient both had to have confidence in a PCP's ability to provide follow‐up. Some oncologists felt that the key to building this trust would be to engage PCPs early on in follow‐up.

In reality, if there are trust issues, maybe knit the primary care provider in early, maybe say half the visits with the primary care provider the first few years, for a couple reasons. One, the patient builds trust. Second, the primary care provider becomes more and more comfortable.

Oncologists' appreciated the relationship that would develop between themselves and a referring PCP; over time, they felt that they could begin to “recognize certain PCP's intuitively that seem to have a good follow‐up, a good rapport.” Oncologists most often determined their level of trust in a provider indirectly by talking to patients about their experiences with the PCP and looking at PCP notes.

So I try to determine, “Is the primary care provider doing a breast exam? Looking for breast issues?” when I talk to the patient. And I look for that in their notes. I don't actually call them up, but if they're not [doing an exam], or there's not evidence of that or the patient tells me no, then I increase my frequency of contact.

Establishing a relationship and trusting the PCP allowed some oncologists to feel comfortable deferring survivorship care.

## Discussion

In this study with breast medical, radiation, and surgical oncologists, we identified a number of oncologist‐perceived challenges to a more expanded role for primary care in breast cancer survivorship care based on oncologists' perceptions of PCPs. These perceptions directly influence oncologists' decision‐making surrounding the follow‐up care they recommend to their patients and likely will impact the implementation of alternative models of survivorship care delivery currently being proposed.

Our findings are similar to those of studies conducted with PCPs. Multiple studies report PCPs' hesitation to assume responsibility for cancer survivorship because of their lack of training in survivorship care and limited practical experience caring for cancer survivors 13, 14, 17, 18, 19. Although many PCPs believe they as a discipline have the skills necessary to “provide follow‐up care related to the effects of cancer or its treatment” (39–59%) and “to initiate appropriate screening or diagnostic work‐up to detect recurrent cancer” (63–75%), a much smaller proportion expressed confidence in their own individual abilities to perform these tasks 13. In a nationally representative sample of PCPs, only 40% expressed confidence about selecting the appropriate tests to evaluate for recurrence and only 23% in their ability to care for late physical effects associated with cancer treatment 13; the level of confidence was substantially smaller when PCP's in a safety‐net hospital were sampled (22% and 14%) 23. Concerns about missing recurrences and the perception that patients receive more reassurance from seeing oncologists contributed to some PCP's preferences for oncology‐led survivorship care 14. Finally, PCPs identified challenges associated with taking on the additional responsibilities for survivorship care within the primary care setting, including limited time to address survivors' cancer needs in addition to their primary care needs and competing clinical priorities 14, 16.

Our findings support the direction of a number of ongoing initiatives to facilitate and expand the role for PCPs in breast cancer survivorship care. Current follow‐up guidelines are written quite generically. More explicit recommendations, such as is outlined in the recently released American Cancer Society/American Society of Clinical Oncology Breast Cancer Survivorship Care Guide, can guide PCPs and are an important step 24. PCPs have also expressed significant support for the concept of survivorship care plans 14, 19, 23, 25, 26. Although limited data exist surrounding the impact of survivorship care plans on patient outcomes, receipt of care plans has been shown to increase PCP confidence in their knowledge about needed survivorship care and expand PCPs' perceptions of their roles in cancer follow‐up 18, 26. Combined, these steps will help both PCPs and oncologists feel confident that all involved parties are aware of the important components of comprehensive follow‐up.

Our findings also suggest that PCPs should be involved in survivorship care early, right after completion of active treatment, to enhance PCP comfort and develop patients' trust in PCPs in this role. Early incorporation would require trust and an ongoing relationship between the oncologist and PCP. Currently, there are relatively limited opportunities for oncologists to build relationships with the PCPs with whom they share patients. A more systematic approach to relationship building between oncologists and PCPs could significantly increase oncologists' willingness to share follow‐up with primary care. This effort would also be aided by increased PCP training in cancer survivorship, either during residency or as part of continuing medical education. Based on the views expressed by oncologists in this analysis, the PCP expertise obtained through additional training would facilitate oncologists' willingness to transfer survivorship care over to PCPs.

It is also clear from our data that a “one size fits all” approach to implementing alternative models of survivorship care is unlikely to be successful. Variability exists surrounding the circumstances where oncologists feel comfortable transitioning responsibility for survivorship care to primary care. As a result of their specialty training and extensive clinical experience caring for breast cancer patients, oncology specialists perceive that they have additional expertise that a generalist may not and that patients at greater risk for recurrence or treatment side‐effects may benefit more from this expertise than patients at lower risk. Because of this, we urge a tailored approach to implementation. Our data suggests that some tailoring of follow‐up already takes place, as oncologists were more likely to continue participating in follow‐up for patients perceived to be high‐risk and more likely to defer follow‐up to primary care for patients in whom oncology follow‐up was perceived to be less valuable (i.e., patients with ductal carcinoma in situ). Additionally, the willingness to share survivorship care with PCPs varies by the type of oncologist involved, with medical oncologists more receptive to an expanded role for PCPs. Selecting higher risk patients and patients for whom there is a role for radiation or surgical oncology involvement for ongoing specialty follow‐up while transitioning patients perceived to be of lower risk or who follow with medical oncology alone to primary care may be one strategy.

Finally, successful implementation of a shared model of survivorship care may require policy level changes to expand the capacity of the primary care system to comprehensively address the needs of breast cancer survivors. Much of the literature promoting alternative models of survivorship care has focused on the *projected* shortfalls in the oncology work force 1, 2, 3 rather than the *current* shortfall in primary care 27, 28, 29. This idea was endorsed by oncologists in our study, who perceived that PCPs' existing workload was burdensome. Based on these perceptions, concerns were raised about whether primary care clinics have the capacity to manage the influx of patients that would result from transitioning follow‐up for the more than 3 million breast cancer survivors in the U.S. to primary care. Furthermore, primary care is currently under‐reimbursed, leading to an increasing emphasis on seeing numerous patients in short time slots and an increasing focus on meeting practice benchmarks. Some form of primary care system redesign may be needed to facilitate the provision of survivorship care in the primary care setting.

This study represents the perspectives of Wisconsin oncologists and may not reflect the perspectives of oncologists practicing across the U.S. However, the complementary nature of our findings with studies conducted outside of Wisconsin suggests that our findings have relevance 12, 13, 14, 17, 18, 19. Furthermore, our use of qualitative methodology means that our findings are not generalizable to the broader population. However, the strengths of qualitative research is that it provides a deeper understanding of individual participants perspectives than can be obtained thought quantitative means 21, 22. This approach best advanced our understanding of oncologists' perceptions of the role PCPs play in breast cancer survivorship care by complimenting the work that already existed in the literature. Finally, this study only reflects the oncologists' perspectives on PCPs ability to provide survivorship care and consequently may not accurately reflect PCPs own perspectives. However, as oncologists are the “gate‐keepers” directing the follow‐up care for their patients, these oncologists perspectives are critical to the implementation of alternative models of survivorship care.

## Conclusions

In this study, we confirmed that Wisconsin oncologists perceive there to be numerous barriers to oncologists sharing survivorship care with PCPs. Although we did not explore the opinions of PCPs in this study, our findings are consistent with those reported within the substantial existing literature examining PCPs perspectives 12, 13, 14, 15, 16, 18. We have identified a number of ongoing initiatives that may support an expanded role for PCPs in providing survivorship care. Additionally, we argued that a tailored approach to follow‐up is likely to be more successful, as a shared model of follow‐up may be perceived as less acceptable for patients at “higher risk” or for whom surgical or radiation oncology follow‐up is felt to be important. The most challenging barrier to address may be the limited capacity of the current primary care system to manage follow‐up for breast cancer survivors.

## Conflict of Interest

Dr. Jacobs serves on an advisory panel for Aetna Insurance Company focused on how to reduce health disparities members. Dr. Tevaarwerk receives research funding (unrelated work) from Takeda and Amgen. Dr. Greenberg has an advisory role for Johnson & Johnson and received research funding from Covidien (unrelated work).
